# Regulation of the NF-κB/NLRP3 signalling pathway by *Shenghui Yizhi* decoction reduces neuroinflammation in mice with Alzheimer’s disease

**DOI:** 10.1080/07853890.2024.2411011

**Published:** 2024-10-11

**Authors:** Peng Wang, Zi-yi Sun, Gao-yu Zhang, Yi Jin, Wei-liang Sun, Bao-sheng Zhao, Xin Chen, Qiu-bing Li

**Affiliations:** a The Second Department of Healthcare, China-Japan Friendship Hospital, Beijing, China; b School of Clinical Medicine, Beijing University of Chinese Medicine, Beijing, China; c Department of Neurology, China-Japan Friendship Hospital, Beijing, China; d Institute of Clinical Medical Sciences, China-Japan Friendship Hospital, Beijing, China; e College of Traditional Chinese Medicine, Beijing University of Chinese Medicine, Beijing, China

**Keywords:** Alzheimer’s disease, neuroinflammation, NF-κB/NLRP3 signalling pathway, *Shenghui Yizhi* decoction, coordinating heart and kidney

## Abstract

**Background:**

*Shenghui Yizhi* Decoction (SHYZD) has exhibited the capacity to enhance cognitive function and learning abilities in individuals diagnosed with Alzheimer’s disease (AD) while ameliorating pre-existing neuroinflammation. Nevertheless, the precise mechanism underlying its therapeutic effects on AD remains to be elucidated.

**Methods:**

Twenty-four male SAMP8 mice were randomly divided into three groups, and eight male SAMR1 mice were used as a blank control, to examine their learning and spatial memory abilities. The expression of amyloid β1-42 (Aβ1-42) was detected by immunohistochemical staining of hippocampal tissue. ELISA was used to detect the interleukin-1β (IL-1β), interleukin-6 (IL-6) and tumour necrosis factor-α (TNF-α) expressions. Real time PCR was used to detect NOD-like receptor thermal protein domain associated protein 3 (NLRP3), cysteine protease-1 (Caspase-1), and IL-1β mRNA expression. Western blot was used to detect nuclear factor kappa-B (NF-κB), inhibitor of NF-κB α (IκBα), IκB kinase α (IKKα), NLRP3, Caspase-1, and IL-1β protein expression.

**Results:**

In this study, SAMP8 mice, employed as an AD model, displayed markedly diminished abilities in terms of spatial localization, navigation, and spatial exploration when compared to the blank control group. Additionally, there was a substantial upregulation of Aβ1-42 expression in the hippocampus of these mice, along with a significant increase in the levels of inflammation-associated factors, including IL-1β, IL-6, TNF-α, NLRP3, Caspase-1, as well as the NF-κB pathway-related proteins, namely, NF-κB, IκBα, and IKKα. Moreover, after treatment with positive drugs (donepezil hydrochloride) and SHYZD, the learning abilities of the mice exhibited significant improvements. Furthermore, the hallmark AD protein Aβ1-42, inflammatory factors, and NF-κB/NLRP3 signalling pathway proteins were significantly reduced. These findings collectively suggest that SHYZD exerts a therapeutic effect on AD.

**Conclusion:**

In summary, the specific molecular mechanisms through which SHYZD alleviates AD and the potential role for SHYZD in the NF-κB/NLRP3 signalling pathway are identified in this study.

## 1. Background

Alzheimer’s disease (AD) is a neurodegenerative disorder characterized by a gradual onset and progressive deterioration and is the leading cause of dementia as per the WHO update [1]. It has swiftly emerged as a significant global public health concern, ranking among the most costly and life-threatening diseases of the current century [2]. An estimated 6.5 million people aged 65 and older in the United States were diagnosed with AD in 2022, and it is now listed as a major cause of death [3]. Both amyloid-β (Aβ), which forms extracellular senile plaques, and hyperphosphorylated tau, which aggregates intracellularly to form neurofibrillary tangles, are elevated in the brain tissue in AD [4]. Numerous studies have demonstrated that a persistent excessive neuroinflammatory response is the primary factor in the progression of AD, which can result in neurodegeneration and disease progression [5]. The neuroinflammatory hypothesis offers fresh insights into unravelling the fundamental mechanisms driving AD. Nuclear factor kappa-B (NF-κB) and NOD-like receptor thermal protein domain-associated protein 3 (NLRP3), recognized as pivotal influencers, are deemed to have significant associations with the progression of AD [6]. Microglia-specific inflammasome activation is a novel pathological mechanism of AD related to neuroimmunoinflammation. Among them, NLRP3 inflammasome, as an important mediator of activation of caspase-1 and IL-1β in the process of innate immunity, is closely related to the occurrence and development of neuroinflammation mechanism of AD [7]. However, the treatment of AD remains problematic due to its complex pathogenesis and limited drug availability. Previous research has demonstrated that NLRP3-related signalling pathway is a key link in the neuroinflammatory response, and inhibition of NLRP3 can enhance the behaviour and pathological damage of AD in transgenic mice, making it a potential therapeutic target for AD [7–9].

*Shenghui Yizhi* decoction (SHYZD) is a traditional Chinese medicine (TCM) prescription for treatment of AD. It was established by Professor Li Qiubing, an expert on geriatrics at China-Japan Friendship Hospital, based on the TCM theory of treating AD as a mental disease. Based on previous clinical studies, it was found that SHYZD can enhance cognitive function and the daily living ability of patients diagnosed with AD [10]. It was also confirmed that SHYZD can improve the learning and memory ­abilities of AD mice in experimental research, but the mechanism remains unknown [11]. Studies have demonstrated that the active ingredients in a traditional Chinese ­medicine decoction have anti-neuroinflammatory effects, which provides support for the potential anti-neuroinflammatory effects of SHYZD [12].

However, traditional drugs cannot effectively treat for AD. SHYZD, as a new alternative therapy, has a promising application for patients. We hypothesize that the herbal compound SHYZD can effectively treat AD by inhibiting the neuroinflammation-related NF-κB/NLRP3 signalling pathway and regulating downstream inflammatory factors.

## 2. Materials and methods

### 2.1. Drugs and reagents

SHYZD consists of 12 herbs (all botanical drugs that make up SHYZD are listed in the Supplementary Material Table 1). The medicinal materials were purchased from Beijing Tongrentang Pharmaceutical Co., Ltd. prepared by the Pharmacy Department of China-Japan Friendship Hospital. Assumed the body weight of an adult man is 70Kg, and thus the dose of SHYZD is 2 g (raw herb)/Kg. In accordance to body surface area conversion between humankinds and mice, the dose of SHYZD is 15.6 g (raw herb)/Kg. The medicinal materials were decocted three times using the traditional water decoction method Specific protocol was as follows: a package of SHYZD was soaked in 1200 g of distilled water for 4 h and decocted for 60 min and finally filtered. The filtrate was decocted in 1200 g of distilled water for 30 min and then filtered through gauze. The step was repeated again. The three filtrate was mixed and concentrated to 115.4 mL. Each 1 mL of the liquid medicine contains 1.04 g of SHYZD.

Donepezil hydrochloride is a long-acting symptomatic treatment for AD and can be used as a positive control for improving memory and cognitive impairment. Donepezil hydrochloride tablets (batch no. 1702010, 5 mg/tablet) was purchased from Eisai China pharmaceutical Co., Ltd. Interleukin-1β (IL-1β) ELISA kits (CRE0006), Interleukin-6 (IL-6) ELISA kits (CRE0005), and Tumour necrosis factor-α (TNF-α) ELISA kits (CRE0003) were purchased from 4 A Biotech. NF-κB p65 antibody(ab8227), NLRP3 antibody(ab16502), Caspase-1 antibody(ab214185), and IL-1β antibody(ab1872) were purchased from Abcam. Reverse Transcription System (A3500) was purchased from Promga. SYBR^®^ Green Realtime PCR Master Mix(QPK-201) was purchased from TOYOBO.

### 2.2. Animals and treatment

SAM-P8 is a kind of senescence-accelerated mouse, whose characters manifest themselves in age-related impairment in memory and the ability to learn and obvious degenerative changes of the central nervous system [11]. Twenty-four SPF SAMP8 male mice and 8 SAMR1 male mice aged 3 months, with body weight of 30 ± 2 g were used in this study. The animals were provided by the Department of Experimental Animal Science, Peking University Health Science Center (No. SCXK, Beijing, 2016-0010). Animals were housed at a temperature of 22 ± 2 °C, humidity of 40%∼70%, and with a 12-hour light-dark cycle. The mice were given unrestricted access to water and food. All animal procedures and protocols were approved by the China-Japan Friendship Hospital Animal Care Committee (zryhyy-21-21-9-21) and conducted in accordance with the “Guiding Principles in the Care and Use of Animals” published by the American Physiological Society.

After acclimating the SAMP8 mice for one week, they were randomly divided into 3 groups (*n* = 8 in each group): the model group, the positive control group (donepezil hydrochloride), and the SHYZD group. Eight SAMR1 mice served as a control group. The equivalent dose for mice was converted according to the body surface area of humans and mice. The dose administered to mice was 9.1 times that of humans. Assumed the body weight of an adult man is 70Kg, and thus the dose of SHYZ is 1.7 g (raw herb)/Kg. The dose of SHYZ donepezil hydrochloride is 0.1 mg/Kg. The SHYZD group was administered 15.6 g/kg SHYZD, the positive control group was administered 1 mg/kg donepezil hydrochloride solution, and the model and the control groups were administered equal volume of double distilled water. The doses were administered *via* gavage at 15 ml/kg once a day for 90 days, and the mice were weighed every 4 weeks.

### 2.3. Morris water maze test

The whole experimental process consisted of positioning navigation and spatial exploration. On the first day, the pool was filled with water until the visible platform rose above the water in the second quadrant, and mice were placed gently in the water facing the pool wall. The tracking device was initiated simultaneously. If the mouse discovered the platform within 60 s, it was allowed to stay on the platform for 5 s; Otherwise, the mouse was placed on the platform for 20 s. The second to fifth day, the experiment focused on positioning navigation: water was added to the pool to submerge the platform to 1 centimeter below the surface. The mice were placed in the water facing the pool wall from one of four center points of the different quadrants. After locating the platform, the mouse rested on the platform for 15 s and then continued the next training from a different location; if the mouse did not find the platform within 60 s, they were guided to the platform by the experimenters and remained on the platform for 15 s; the escape latency was recorded as 60 s. Training was repeated four times for each mouse, with each mouse being put into the water from four starting points of different quadrants in each time. The tracking device recorded escape latency, swimming path, the time spent in each quadrant and other parameters of the mouse in locating the platform. The average latency of four times was considered as the learning performance of the mouse. On the sixth day, the experiment focused on spatial exploration: the platform in the second quadrant was removed, and the mice were placed in water facing the pool wall from the same starting positions. The swimming path, the number of times of crossing the platform position, and the time spent in the second quadrant within 60 s were recorded to measure the spatial positioning ability of the mice. The mice were monitored by a fixed camera directly above the pool and Ethovision 3.0 software was used to measure the parameters.

### 2.4. Immunohistochemistry

After the last behavioural test, mice were anesthetized with isoflurane. The brain was extracted through craniotomy following cardiac perfusion and fixation. The brain tissue was subsequently encased in paraffin and sectioned into slices measuring 5 μm in thickness. These sections were subsequently subjected to deparaffinization and hydration processes. High-pressure antigen retrieval was carried out using a citrate buffer (pH = 6.0). The sections were treated with 3% hydrogen peroxide in methanol for 15 min to inhibit the endogenous peroxidase, followed by an incubation with normal serum to prevent any non-specific staining. At 4 °C, sections were incubated overnight with rabbit anti-amyloid β1-42 (Aβ1-42). After washing, the tissue sections were treated with the biotinylated anti-rabbit secondary antibody, followed by additional incubation with the streptavidin-horseradish peroxidase complex. After staining with the diaminobenzidine kit, the sections were counterstained with hematoxylin. A negative control was treated simultaneously with the same procedure, but phosphate-buffered saline was used instead of the primary antibody. In the CA1 region of the hippocampus, three successively distinct visual fields were chosen from each slice. The integral optical density and area of each image were captured and analyzed by the Image-ProPlus 6.0 software, and the mean optical density (MOD) was calculated by dividing integral optical density by area.

### 2.5. ELISA

The mice were sacrificed after the behavioural tests. The hippocampal tissue was quickly removed from each mouse and stored in a freezer at −80 °C for further analysis. Expression levels of IL-1β, IL-6, and TNF-α in the hippocampal tissue were determined using ELISA kits based on the protocol provided in the kit. The concentration was set based on the manual of the kit and was calculated with reference to the standard curve.

### 2.6. Reverse transcription polymerase chain reaction (RT-PCR)

TRIzol was used to extract total RNA from hippocampal tissue. In accordance with the manufacturer’s instructions, RNA was reverse-transcribed into cDNAs using the TransScript RT kit. Real-time PCR was used to conduct quantitative PCR. In order to amplify DNA, the reaction conditions were 94 °C for 3 min, followed by 40 cycles of 94 °C for 30 s, 60 °C for 30 s, and 72 °C for 45 s. Based on the 2 − ΔΔCt method, the PCR data was normalized to the level of β-actin gene expression and the value was calculated. Real-time quantitative PCR primers were acquired from Shanghai Shenggong Biological Engineering Technology Service Co., Ltd. The primers are exhibited in Supplementary Material Table 2.

### 2.7. Western blot analysis

The hippocampal tissue was homogenized in radioimmunoprecipitation assay lysis buffer (RIPA lysis buffer, containing protease inhibitor), then centrifuged at 12,000 ×g for 15 min, and the protein concentration was determined using Coomassie-blue reagent. Subsequently, protein extracts from different groups were separated by SDS-polyacrylamide gel electrophoresis and transferred to a polyvinylidene fluoride (PVDF) membrane. The PVDF membrane was blocked with 5% skim milk at room temperature for 2 h before being incubated overnight at 4 °C with the respective primary antibodies. After washing with Tris Buffered Saline Tween three times, the PVDF membrane was incubated with the HRP-conjugated secondary antibodies. The primary antibodies used were IKKα, IκBα, NF-κB, NLRP3, Caspase-1, and IL-1β. Subsequently, the developed bands were visualized by enhanced chemiluminescence advanced kit and gel imaging system. β-acting was used as internal reference. The grayscale values of the bands were analyzed using Image J software (National Institutes of Health).

### 2.8. Statistical analysis

Graphs were prepared and the statistical data were analyzed using GraphPad software (version 8.0, GraphPad Software, Inc., La Jolla, CA, USA) and SPSS Statistics software(version 25.0, IBM., Armonk, NY, USA). Continuous variables were presented as the mean values ± standard deviation (SD). If data showed a normal distribution, Student’s t-test was used to assess the differences between the two groups; if not, the Mann–Whitney test was utilized. *P*-value <0.05 was considered to indicate a statistically significant difference, *n* = 3.

## 3. Results

### 3.1. Positioning navigation experiments

**Figure 1.**
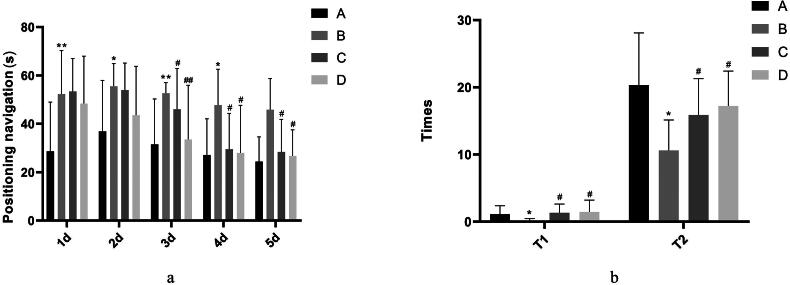
Learning and cognition in experimental mice. a. Comparison of positioning navigation experiments in each group. b. a comparison of spatial exploration capabilities in each group. (A) The control group, (B) the model group, (C) the positive control group (donepezil hydrochloride), and (D) the SHYZD group. Compared with the control group, *P < 0.05, **P < 0.01, n = 3; Compared with the model group, ^#^*P* < 0.05, ^##^*P* < 0.01, n = 3.

The escape latency of mice in the model group was prolonged when compared with the control group (*p* < 0.05). The escape latency of mice in the positive control group (donepezil hydrochloride) and SHYZD group was shortened from the 3^rd^ day when compared with the model group (*p* < 0.05 or *p* < 0.01) (Figure 1a, Supplementary Material Table 3).

### 3.2. Spatial exploration ability

The mice in the model group spent less time in the target quadrant, and the times of crossing the platform decreased when compared with the control group (*p* < 0.05). Mice in the positive control group (donepezil hydrochloride) and SHYZD group took longer to cross the platform and remain in the target quadrant when compared with the model group (*p* < 0.05, Figure 1b, Supplementary Material Table 4).

### 3.3. Hippocampal Aβ1-42 protein expression

**Figure 2.**
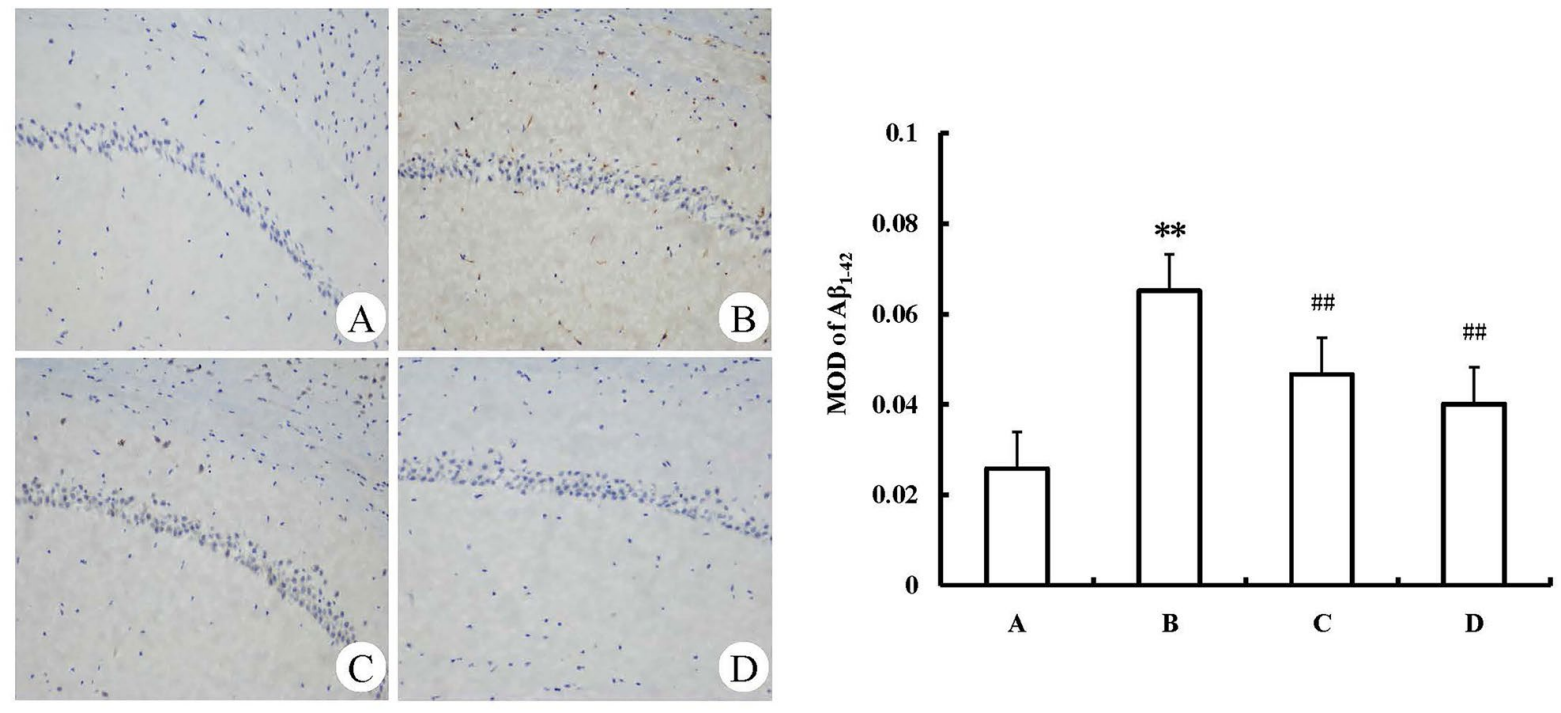
Aβ1-42 protein expression levels in the hippocampal CA1 region (immunohistochemistry, ×200). (A) The control group; (B) The model group; (C) The positive control group (donepezil hydrochloride); (D) The SHYZD group; (E) Compared with the control group, **P < 0.01; Compared with the model group, ^##^*P* < 0.01, n = 3.

The expression of Aβ1-42 protein in the hippocampus of the model group was increased when compared with the control group (*p* < 0.01). The expressions of Aβ1-42 in the positive control group (donepezil hydrochloride) and SHYZD group were reduced, and the differences were statistically significant when compared with the model group (*p* < 0.01, Figure 2).

### 3.4. Hippocampal inflammatory factor expression

**Figure 3.**
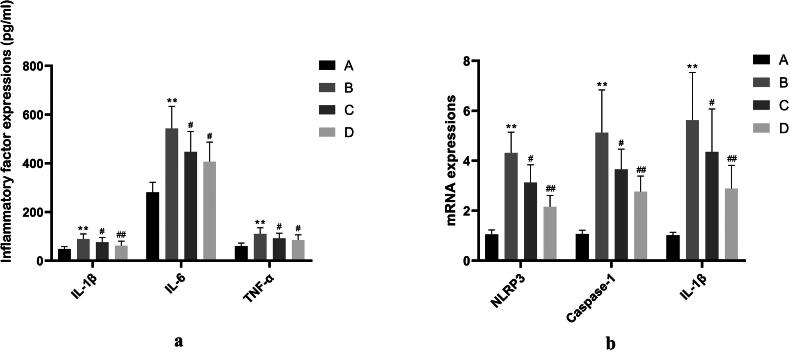
Hippocampal inflammatory factor expression. a. Comparison of hippocampal IL-1β, IL-6, and TNF-α content in each group. b. comparison of hippocampal NLRP3, Caspase-1, and IL-1β mRNA expressions in each group. (A) The control group; (B) The model group; (C) The positive control group (donepezil hydrochloride); (D) The SHYZD group. Compared with the control group, *P < 0.05, **P < 0.01, n = 3; Compared with the model group, ^#^*P* < 0.05, ^##^*P* < 0.01, n = 3.

The levels of IL-1β and IL-6 in the model group were significantly increased when compared with the control group (*p* < 0.01). The IL-1β, IL-6, and TNF-α content in the positive control group and SHYZD group were significantly reduced when compared with the model group (*p* < 0.01 or *p* < 0.05, Figure 3a, Supplementary Material Table 5).

### 3.5. mRNA expression of hippocampal NLRP3-related inflammatory factors

The expressions of NLRP3, Caspase-1 and IL-1β mRNA in the hippocampus of the model group were elevated when compared with the control group (*p* < 0.01). The expressions of hippocampal NLRP3, Caspase-1, and IL-1β mRNA in the positive control group and SHYZD group were reduced when compared with the model group (*p* < 0.01 or *p* < 0.05, Figure 3b, Supplementary Material Table 6).

### 3.6. Hippocampal NF-κB/NLRP3 signaling pathway factor expression

**Figure 4.**
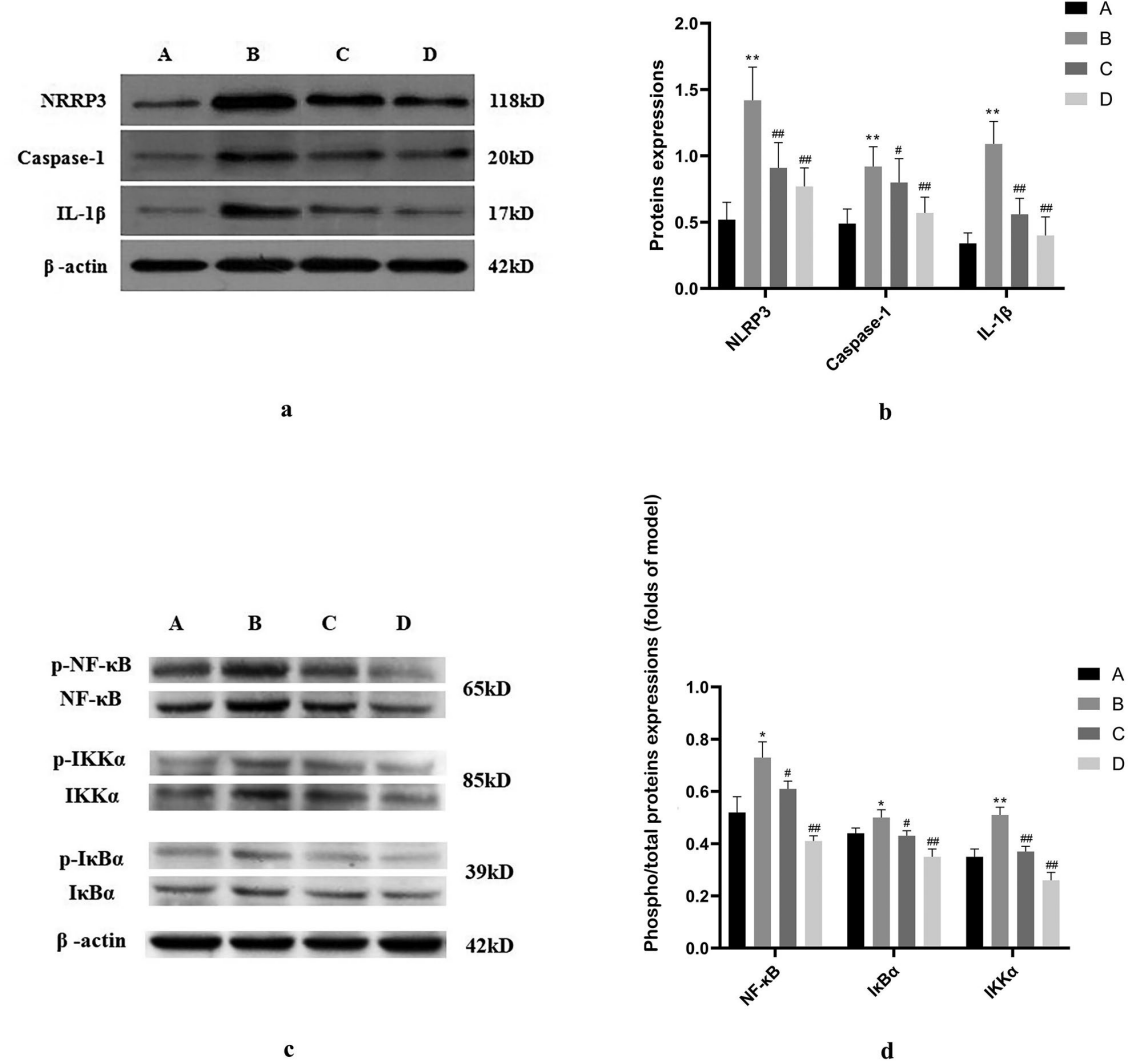
Hippocampal NF-κB/NLRP3 signalling pathway factor expression. a-b. Expressions of hippocampal NLRP3, Caspase-1, and IL-1β proteins in each group. c-d. Expressions of hippocampal NF-κB, IκBα, and IKKα proteins in each group. (A) The control group; (B) The model group; (C) The positive control group (donepezil hydrochloride); (D) The SHYZD group. Compared to the control group, *P < 0.05, **P < 0.01, n = 3; Compared with the model group, ^#^*P* < 0.05, ^##^*P* < 0.01, n = 3.

The expressions of hippocampal NLRP3, Caspase-1, and IL-1β proteins in the model group were elevated when compared with the control group (*p* < 0.01). The expressions of hippocampal NLRP3, Caspase-1, and IL-1β proteins in the positive control group and SHYZD group were decreased when compared with the model group (*p* < 0.01 or *p* < 0.05, Figure 4a-b, Supplementary Material Table 7).

Similarly, related marker proteins were identified in the NF-κB/NLRP3 pathway. The expressions of hippocampal NF-κB, IκBα, and IKKα proteins in the model group were increased when compared with the control group (*p* < 0.01 or *p* < 0.05). The expressions of hippocampal NF-κB, IκBα, and IKKα proteins in the positive control group and SHYZD group were reduced when compared with the model group (*p* < 0.01 or *p* < 0.05, Figure 4c-d, Supplementary Material Table 8). In addition, phosphorylated protein expression was measured, and a consistent pattern emerged (Figure 4c).

## 4. Discussion

In accordance with traditional Chinese medicine, AD is categorized within the domain of forgetfulness, dementia, and related disorders. Ancient physicians predominantly expounded upon the etiology and pathogenesis of AD in terms of various factors, including essence deficiency and marrow depletion, qi and blood insufficiency, blockage of phlegm and blood stasis in the orifices, and the obstruction of collaterals by turbid toxins [13–15]. Professor Li Qiubing has been devoted to the prevention and treatment of AD with traditional Chinese medicine for many years. As a neurodegenerative disease manifested primarily by memory and intelligence disorders and non-normal mental behaviour, he believes that AD falls under the category of mental disease in traditional Chinese medicine.

TCM, single or compounded, have a long history of use in the treatment of neurodegenerative diseases. For example, SHYZD was modified based on the *Shenghui* Decoction developed by Chen Shiduo in the Qing Dynasty. The prescription comprises Shu Dihuang, Shan Zhuyu, and Tu Sizi, substances for which pharmacological investigations have demonstrated cognitive-enhancing, anti-inflammatory, and neuroprotective properties [16, 17]. The bioactive constituents present in Ren Shen, Mai Dong, Fu Shen, Bai Ziren, and Suan Zaoren have been observed to augment the expression of NMDA receptors and synaptic functional proteins, thereby ameliorating the manifestations of learning and memory impairments in rTg4510 mice [18]. The active components within Yuan Zhi, Chang Pu, and Bai Jiezi exhibit pharmacological attributes encompassing anti-anxiety, anti-dementia effects, the enhancement of synaptic plasticity, and the facilitation of nerve cell differentiation and regeneration [18–20]. The efficacy of this compound formula has been substantiated by prior clinical and foundational investigations [10, 11].

The precise pathogenesis of Alzheimer’s disease (AD) remains elusive. However, a multitude of studies have provided evidence that the neuroinflammatory response plays a pivotal role in the development of AD. It is regarded as the third fundamental pathological alteration, following the deposition of Aβ (amyloid-beta) and the formation of neurofibrillary tangles, contributing significantly to the disease process [21]. Aβ deposition can induce a neuroinflammatory response by activating microglia, leading to the release of inflammatory mediators and mediating neurotoxicity [22]. In turn, excessive inflammatory factors can increase the production of Aβ to form a malignant inflammatory cascade, ultimately accelerating the progression of AD [23]. Few specific treatments target the underlying pathological mechanisms. The treatment of AD focuses primarily on balancing neurotransmitter disorders and alleviating disease symptoms [24]. Currently, the most widely used drugs for the treatment of AD consist of cholinesterase inhibitors and N-methyl-D-aspartic acid receptor antagonists, which can temporarily alleviate the symptoms, but are frequently accompanied by severe side effects, lack disease-modifying efficacy, and some patients are prone to drug resistance [25]. We investigated the specific molecular mechanism *in vivo* and assessed the effect of this drug on the NF-κB/NLRP3 signalling pathway, to provide new insights for the intervention of SHYZD on AD from the perspective of inhibiting neuroinflammatory response.

NLRP3 is a sensor component of NLRP3 inflammasomes and plays a crucial role in their subsequent formation and activation [26]. In recent years, the importance of NLRP3 inflammasome in the pathogenesis of AD has become increasingly significant, and is now considered to be the core mechanism of AD inflammatory response [27]. NLRP3 is highly expressed in microglia. Upon activation by Aβ, it forms NLRP3 inflammasome, a high molecular weight protein complex with apoptosis-associated speck-like protein containing a CARD (ASC) and Caspase-1 precursor, which leads to the activation of Caspase-1 and promotes the maturation and secretion of IL-1β [28]. In the extracellular milieu, IL-1β is released and engages with IL-1β receptors situated on neurons, subsequently facilitating neuronal apoptosis. Conversely, when IL-1β binds to receptors on microglial cells, it triggers a sustained activation of microglia, resulting in the generation of an abundance of pro-inflammatory factors, including TNF-α, IL-6, IL-18, and IL-1β itself. This pro-inflammatory milieu collectively contributes to neuronal damage and degeneration [29]. NF-κB is a key regulator of inflammatory cytokine production [30], and its activation is a key target in the inflammatory cascade of AD [31]. In a priming step, transcriptionally active signalling receptors induce the NF-kB-dependent induction of NLRP3 itself as well as that of the Caspase-1 substrates of the pro-IL-1β family [32]. In AD, activated NF-κB is primarily distributed in neurons and glial cells surrounding Aβ plaques, and is closely associated with synaptic plasticity and memory process [32, 33]. Cascade activation of the NF-κB signalling pathway is related to the expression of AD inflammatory factor and the level of neuroinflammation [34, 35]. It is believed that traditional Chinese medicine can regulate the signalling pathways associated with the pathologic progression of AD, including the NF-κB signalling pathway [11, 36]. Therefore, we hypothesize that NLRP3 inflammasome-mediated cascade may be one of the mechanisms by which SHYZD exerts therapeutic effects on AD, and SHYZD may potentially regulate NF-κB-related pathways.

Nowadays, SAM-P8 mouse is a model widely used for AD, aging and analysis of relevant drugs [37–40]. Our studies indicates that SAM-P8 mouse show a decline in memory under Morris Water Maze Test. Meanwhile, SAM-P8 mouse were accompanied by the abnormal deposition of Aβ1-42 prefrontal cortices and the hippocampi.

The levels of inflammatory factors TNF-α, IL-6, and IL-1β were elevated in the hippocampus of SAMP8 mice compared with SAMR1 mice, suggesting that the interaction between the inflammatory response and Aβ deposition lead to the impairment of learning and memory. Simultaneously, the expression levels of hippocampal NLRP3, Caspase-1, IL-1β mRNA and protein were significantly increased, further indicating that the NLRP3/Caspase-1/IL-1β pathway-mediated neuroinflammatory response was involved in the incidence and progression of AD. Subsequent to treatment with SHYZD, a notable enhancement in learning and memory capabilities was observed in SAMP8 mice. Additionally, there was a marked reduction in the levels of inflammatory factors, namely TNF-α, IL-6, and IL-1β, within the hippocampus. Furthermore, the expression levels of key molecules associated with the NLRP3 inflammasome pathway, including NLRP3, Caspase-1, IL-1β mRNA, and protein, exhibited significant decreases. Additionally, alterations were noted in the expressions of NF-κB, IκBα, and IKKα, constituents of the NF-κB-related pathway. These findings collectively suggest that SHYZD may enhance learning and memory abilities in AD by modulating the NF-κB/NLRP3 pathway to inhibit inflammasome activation and mitigate the neuroinflammatory response.

## 5. Conclusion

Our studies demonstrated that SHYZD effectively improved the learning and cognitive abilities of mice while concurrently reducing the expression of Aβ1-42 protein. Furthermore, our findings suggest that SHYZD exerts its therapeutic effects on AD by modulating neuroinflammatory processes. Notably, this regulation appears to involve the NF-κB/NLRP3 signalling pathway, indicating a potential mechanism of action for SHYZD in AD treatment. However, the biological activities and metabolic pathways of these components entering the blood, especially the brain, will be further investigated, which will provide experimental basis for better elucidating the pharmacodynamic substances and mechanisms of SHYZD in the treatment of AD *in vivo.*

## Ethical approval

All animal procedures and protocols were approved by the China-Japan Friendship Hospital Animal Care Committee (zryhyy-21-21-9-21) and conducted in accordance with the “Guiding Principles in the Care and Use of Animals” published by the American Physiological Society. All applicable international, national, and/or institutional guidelines for the care and use of animals were followed. We have adhered to ARRIVE guidelines to proceed.

## Consent for publication

Not applicable.

## Supplementary Material

SUPPLEMENTARY MATERIALS.doc

## Data Availability

The datasets used and/or analyzed during the current study are available from the corresponding author upon reasonable request.
