# Supplementary material for: Regulation of the NF-κB/NLRP3 signalling pathway by Shenghui Yizhi decoction reduces neuroinflammation in mice with Alzheimer’s disease
Source: Ann Med. 2024 Oct 11;56(1):2411011. doi: 10.1080/07853890.2024.2411011 (PMC11486153; doi:10.1080/07853890.2024.2411011)
Supplement: SUPPLEMENTARY MATERIALS.doc [file IANN_A_2411011_SM2394.doc]

**Supplementary Material Table 1. Components of SHYZD (intervention drug)**

| Chinese name | Chinese Pinyin | Scientific name | Family | Part used | Daily dosage(g) |
| --- | --- | --- | --- | --- | --- |
| 熟地黄 | Shu Dihuang | Rehmannia glutinosa (Gaertn.) DC. | Orobanchaceae | steamed root | 15 |
| 山茱萸 | Shan Zhuyu | Cornus officinalis Siebold & Zucc. | Cornaceae | dried ripe sarcocarp | 12 |
| 菟丝子 | Tu Sizi | Cuscuta chinensis Lam. | Convolvulaceae | dried ripe seed | 12 |
| 人参 | Ren Shen | Panax ginseng C.A.Mey. | Araliaceae | dried root | 9 |
| 麦冬 | Mai Dong | Ophiopogon japonicus (Thunb.) Ker Gawl. | Asparagaceae | dried root tuber | 9 |
| 茯神 | Fu Shen | Smilax glabra Roxb. | Smilacaceae | dried rhizome | 12 |
| 白术 | Bai Zhu | Atractylodes macrocephala Koidz. | Asteraceae | dried rhizome | 9 |
| 柏子仁 | Bai Ziren | Platycladus orientalis (L.) Franco | Cupressaceae | seed | 9 |
| 酸枣仁 | Suan Zaoren | Ziziphus jujuba Mill. | Rhamnaceae | dried ripe fruit | 9 |
| 远志 | Yuan Zhi | Polygala tenuifolia Willd. | Polygalaceae | dried root | 9 |
| 菖蒲 | Chang Pu | Acorus calamus L. | Acoraceae | dried rhizome | 9 |
| 白芥子 | Bai Jiezi | Sinapis alba L. | Brassicaceae | ripe seed | 6 |

**Supplementary Material Table 2. Primer sequence**

| **Gene** | **Primer Sequence (5’-3’)** | **Length/bp** |
| --- | --- | --- |
| NLRP3 | Forward GCTGCTCAGCTCTGACCTCT | 108 |
|  | Reverse GTCCCTCACAGAGGAGCTTG |  |
| Caspase-1 | Forward TATGGAAAAGGCACGAGACC | 137 |
|  | Reverse CAGCTGATGGACCTGACTGA |  |
| IL-1β | Forward AGGCTTCCTTGTGCAAGTGT | 220 |
|  | Reverse TGAGTGACACTGCCTTCCTG |  |
| β-actin | Forward CCCATCTATGAGGGTTACGC | 150 |
|  | Reverse TTTAATGTCACGCACGATTTC |  |

**Supplementary Material Table 3. Comparison of positioning navigation experiments in each group（x±s）(n=8)**

| **Group** | **Positioning Navigation（s）** | | | | |
| --- | --- | --- | --- | --- | --- |
|  | **1d** | **2d** | **3d** | **4d** | **5d** |
| A | 28.65±20.37 | 36.45±21.55 | 31.50±18.38 | 27.13±14.88 | 24.38±10.31 |
| B | 52.38±17.84^**^ | 55.00±9.49^*^ | 52.75±4.92^**^ | 47.75±14.86^*^ | 45.75±12.84 |
| C | 52.75±13.30 | 54.01±11.11 | 46.03±16.90^#^ | 29.50±14.24^#^ | 28.38±13.44^#^ |
| D | 48.38±19.63 | 43.75±19.60 | 33.03±22.41^##^ | 28.00±19.90^#^ | 26.75±10.43^#^ |

(A) The control group; (B) The model group; (C) The positive control group (Donepezil hydrochloride); (D) The SHYZD group Compared with the control group, ^*^*P<*0.05, ^**^*P<*0.01; Compared with the model group, ^#^*P<*0.05, ^##^*P<*0.01

**Supplementary Material Table 4. Comparison of space exploration ability in each group（x±s）(n=8)**

| **Group** | **The times of crossing the platform within 60s** | **The residence time in the target quadrant** |
| --- | --- | --- |
| A | 1.13±1.25 | 20.34±7.76 |
| B | 0.13±0.35^*^ | 10.61±4.54^*^ |
| C | 1.33±1.30^#^ | 15.89±5.41^#^ |
| D | 1.46±1.76^#^ | 17.23±5.19^#^ |

(A) The control group; (B) The model group; (C) The positive control group (Donepezil hydrochloride); (D) The SHYZD group

Compared with the control group, ^*^*P<*0.05, ^**^*P<*0.01; Compared with the model group, ^#^*P<*0.05

**Supplementary Material Table 5. Comparison of hippocampal IL-1β, IL-6, and TNF-α contents in each group（pg/g, x±s）(n=8)**

| **Group** | **IL-1β** | **IL-6** | **TNF-α** |
| --- | --- | --- | --- |
| A | 48.38±10.06 | 280.84±41.36 | 60.41±12.29 |
| B | 89.74±20.31^**^ | 543.66±90.52^**^ | 110.55±25.13^**^ |
| C | 76.29±19.65^#^ | 447.51±83.23^#^ | 92.76±20.71^#^ |
| D | 62.21±18.46^##^ | 406.95±80.69^#^ | 85.21±21.43^#^ |

(A) The control group; (B) The model group; (C) The positive control group (Donepezil hydrochloride); (D) The SHYZD group

Compared with the control group, ^*^*P<*0.05, ^**^*P<*0.01; Compared with the model group, ^#^*P<*0.05, ^##^*P<*0.01

**Supplementary Material Table 6. Comparison of hippocampal NLRP3, Caspase-1, and IL-1β mRNA expressions in each group（pg/g, x±s）(n=8)**

| **Group** | **NLRP3** | **Caspase-1** | **IL-1β** |
| --- | --- | --- | --- |
| A | 1.05±0.18 | 1.07±0.15 | 1.02±0.12 |
| B | 4.32±0.83^**^ | 5.13±1.71^**^ | 5.63±1.91^**^ |
| C | 3.13±0.71^#^ | 3.66±0.81^#^ | 4.36±1.72^#^ |
| D | 2.16±0.45^##^ | 2.77±0.62^##^ | 2.89±0.93^##^ |

(A) The control group; (B) The model group; (C) The positive control group (Donepezil hydrochloride); (D) The SHYZD group

Compared with the control group, ^*^*P<*0.05, ^**^*P<*0.01; Compared with the model group, ^#^*P<*0.05, ^##^*P<*0.01

**Supplementary Material Table 7. Comparison of hippocampal NLRP3, Caspase-1 and IL-1β proteins expression in each group（x±s）(n=8)**

| **Group** | **NLRP3** | **Caspase-1** | **IL-1β** |
| --- | --- | --- | --- |
| A | 0.52±0.13 | 0.49±0.11 | 0.34±0.08 |
| B | 1.42±0.25^**^ | 0.92±0.15^**^ | 1.09±0.17^**^ |
| C | 0.91±0.19^##^ | 0.80±0.18^#^ | 0.56±0.12^##^ |
| D | 0.77±0.14^##^ | 0.57±0.12^##^ | 0.40±0.14^##^ |

(A) The control group; (B) The model group; (C) The positive control group (Donepezil hydrochloride); (D) The SHYZD group

Compared with the control group, ^*^*P<*0.05, ^**^*P<*0.01; Compared with the model group, ^#^*P<*0.05, ^##^*P<*0.01

**Supplementary Material Table 8. Comparison of hippocampal NF-κB, IκBα and IKKα proteins expression in each group（x±s）(n=8)**

| **Group** | **NF-κB** | **IκBα** | **IKKα** |
| --- | --- | --- | --- |
| A | 0.52±0.06 | 0.44±0.02 | 0.35±0.03 |
| B | 0.73±0.06^*^ | 0.50±0.03^*^ | 0.51±0.03^**^ |
| C | 0.61±0.03^#^ | 0.43±0.02^#^ | 0.37±0.02^##^ |
| D | 0.41±0.02^##^ | 0.35±0.03^##^ | 0.26±0.03^##^ |

(A) The control group; (B) The model group; (C) The positive control group (Donepezil hydrochloride); (D) The SHYZD group

Compared with the control group, ^*^*P<*0.05, ^**^*P<*0.01; Compared with the model group, ^#^*P<*0.05, ^##^*P<*0.01
